# Refining the prognostic understanding of recurrence in hepatic cystic echinococcosis surgery: a matters arising perspective

**DOI:** 10.1186/s41182-025-00872-9

**Published:** 2025-12-16

**Authors:** Kashf Younas, Raghabendra Kumar Mahato

**Affiliations:** 1 Bakhtawar Amin Medical and Dental College, Multan, Pakistan; 2 Gandaki Medical College Teaching Hospital and Research Center, Pokhara, Nepal

**Keywords:** Hepatic cystic echinococcosis, Surgical outcomes, Methodological critique, Disease recurrence, Prognostic factors

## Abstract

This Matters Arising article provides a methodological commentary on the recent study by Rivadeneira et al. concerning the impact of hepatic cystic echinococcosis (HCE) recurrence on postoperative outcomes. While acknowledging the importance of their findings from an endemic region of Chile, we aim to critically examine key methodological aspects to clarify the independent prognostic role of recurrence. We discuss considerations including potential residual confounding from unadjusted cyst characteristics and surgical variables, the ascertainment of recurrence based on imaging, and the generalizability of the results from a high-endemicity population. The core purpose is to build upon this valuable work by proposing methodological refinements for future research. We recommend multi-center studies, standardized outcome sets, and combined follow-up protocols to precisely quantify the burden of HCE recurrence and enhance the global applicability of the findings for improved clinical guidelines.

To the Editor,

I read with great interest the recent retrospective cohort study by Rivadeneira et al. (“Impact of recurrence of hepatic cystic echinococcosis on postoperative outcomes in an endemic region of Chile: a retrospective cohort study”, Tropical Medicine and Health, 2025, 53:153). The authors provide a valuable analysis of a significant clinical challenge, demonstrating that recurrence of hepatic cystic echinococcosis (HCE) is associated with a fivefold increased risk of postoperative complications (POC). This work usefully quantifies a key surgical risk in an endemic region, contributing to the scarce literature on HCE outcomes in Latin America.

The study's strengths include its long-term follow-up, application of robust regression techniques, and adherence to STROBE guidelines. Notwithstanding these strengths, we wish to examine several methodological aspects to clarify the independent prognostic role of recurrence and strengthen the generalizability of the findings. The following discussion aims to build upon their important work by examining key methodological assumptions that, if addressed in future research, could enhance the precision and clinical applicability of such prognostic assessments.

## Evaluating key methodological considerations

### Adjustment for confounding factors

The study identifies recurrence as an independent risk factor for POC after adjusting for evolutionary complications. While this adjustment is crucial, the multivariable model was necessarily limited in the number of covariates included due to the low number of outcome events. Several established prognostic factors for poor surgical outcomes in HCE, such as cyst characteristics (e.g., specific WHO classification stages), surgical technique detail (e.g., precise type of radical procedure, use of scolicidal agents), and perioperative albendazole therapy, were not included in the final adjusted model for POC [5, 7].

Rationale for scrutiny: The omission of these well-documented confounders means the reported adjusted relative risk (RR) of 5.1 may overestimate the independent effect of recurrence. Complex cyst stages (CE3b, CE4, CE5) are independently associated with both higher recurrence risk and increased POC rates [5]. If such cysts were more prevalent in the recurrence group, they could substantially confound the observed association. Future studies with larger cohorts would benefit from a more comprehensive adjustment model incorporating these factors to isolate the true effect of recurrence.

### Definition and ascertainment of recurrence

The study defined recurrence as “the appearance of new active intra-abdominal cysts following surgical intervention”, confirmed by abdominal ultrasound. This represents a standard and clinically relevant definition. However, the sensitivity of ultrasound for detecting early recurrence or small new cysts can be variable, potentially leading to outcome misclassification [4].

Rationale for scrutiny: While non-differential misclassification typically biases results toward the null, the rigorous 60 month follow-up requirement for the primary HCE group might create ascertainment imbalance. The recurrence group, by nature of their clinical presentation, had confirmed disease, while the primary group’s non-recurrence status depended on imaging surveillance sensitivity. Incorporating serological follow-up (e.g., specific IgG antibodies) alongside imaging could improve detection sensitivity in future prospective studies, providing more nuanced outcome assessment [4].

### Generalizability from a highly endemic sub-region

The study was conducted in Chile's La Araucanía region, which demonstrates notably high HCE incidence (up to 6/100,000) compared to national averages [2]. The cohort exhibited a high prevalence of evolutionary complications (72.7%) and substantial recurrence rates (27.9%).

Rationale for scrutiny: These characteristics indicate a population with advanced disease burden [2, 3]. While immensely valuable for this high-endemicity context, the findings may not fully generalize to settings with lower incidence rates, less advanced disease presentation, or different surgical protocols. The magnitude of associated risk (RR = 5.1) might differ in populations where primary surgery addresses simpler, uncomplicated cysts. Acknowledging this context-dependency is crucial for appropriate extrapolation to other endemic regions.

### Analysis of length of hospital stay (LHS)

The study found a one-day longer LHS in the recurrence group in univariate analysis, though this association lost significance in robust linear regression models. The authors appropriately note the low explained variance (adjusted R^2^) and presence of outliers.

Rationale for scrutiny: The non-significant finding despite clinical difference underscores LHS’s complexity as an outcome metric. Factors beyond recurrence status—including complication severity, institutional protocols, and socioeconomic considerations affecting discharge—likely drive most variability. Future analyses might employ zero-truncated or Poisson regression for count data like LHS, or focus on LHS specifically among those experiencing POC [6].

### Handling of mortality data

The study reported a non-significant trend toward higher mortality in the recurrence group (7.0% vs. 2.7%, RR: 2.6). The low event count precluded adjusted analysis.

Rationale for scrutiny: This limitation is understandable in single-center surgical studies of relatively rare conditions. However, the clinically substantial 2.6-fold risk increase should be interpreted as a potentially important signal rather than a definitive negative finding. This trend strongly supports the authors’ recommendation for multi-center collaboration to achieve sufficient power for investigating this critical patient-centered outcome [1].

## Recommendations for future research

To build upon the foundational evidence provided by Rivadeneira et al., we propose these methodological refinements for future studies quantifying HCE recurrence’s independent burden:

**Table:** 

Recommendations	Rationale
1. Conduct multi-center collaborative studies with standardized data collection	Would yield larger sample sizes, enabling more comprehensive adjustment for confounders and a powerful analysis of rare outcomes like mortality
2. Implement a core outcome set including detailed cyst classification, surgical techniques, and perioperative medical therapy	Would minimize residual confounding and facilitate more valid comparisons and meta-analyses across different study populations
3. Utilize combined imaging and serological follow-up protocols for recurrence detection	Would improve the accuracy and sensitivity of recurrence ascertainment, reducing outcome misclassification bias [4]
4. Perform stratified analyses or meta-analyses across different endemicity settings	Would help clarify how the burden of recurrence varies with regional disease prevalence and clinical presentation, enhancing generalizability [2]
5. Investigate patient-reported outcomes and long-term quality of life	Would provide a more holistic understanding of the true impact of recurrence beyond traditional clinical endpoints

## Conclusion

Rivadeneira and colleagues have significantly advanced our understanding by rigorously demonstrating the association between HCE recurrence and postoperative complications in a high-endemicity setting. The methodological considerations discussed here aim to complement their work by outlining pathways for future research to refine these important findings. Implementing more granular adjustment strategies, multi-center designs, and sensitive outcome measures will enable more precise delineation of recurrence’s independent prognostic role. Such methodological refinement is essential for translating this valuable regional evidence into globally applicable clinical guidelines and optimized surgical management strategies for this neglected tropical disease.

## Data Availability

No datasets were generated or analysed during the current study.
